# Myricetin alleviated hydrogen peroxide-induced cellular senescence of nucleus pulposus cell through regulating SERPINE1

**DOI:** 10.1186/s13018-022-03463-0

**Published:** 2023-02-27

**Authors:** Rongsheng Chen, Xiaobo Zhang, Xitian Zhu, Changsheng Wang, Weihong Xu

**Affiliations:** 1 Department of Spinal Surgery, The First Affiliated Hospital of Fujian Medical University, No. 20 Chazhong Road, Fuzhou, 35005 Fujian China; 2 Department of Spinal Surgery, National Regional Medical Center, Binhai Campus of the First Affiliated Hospital, Fujian Medical University, 350212 Fuzhou, China

**Keywords:** Myricetin, Senescence, Nucleus pulposus cells, SERPINE1

## Abstract

**Background:**

Myricetin (MYR) is a common plant flavonoid with antioxidant and anticancer properties. However, the anti-aging effect of MYR on nucleus pulposus cells (NPCs) is still unknown. The study aimed to explore the effect of MYR on the senescence of NPCs.

**Methods:**

Methyl-thiazolyl tetrazolium assay was used to detect NPCs viability. Senescence level was evaluated by senescence-associated β-galactosidase (SA-β-Gal) staining and the expression levels of P21, P16, IL-6 and IL-8. RNA-Sequencing (RNA-seq) technology was used to identify differentially expressed genes (DEGs) between hydrogen peroxide + MYR (HO + MYR) group and HO group, and Gene Ontology (GO) functional was performed to analyze DEGs. A Venn diagram was generated to screen overlapping DEGs related to aging and inflammation, and the role of the promising validated DEG was selected for further investigation by gene functional assays.

**Results:**

HO inhibited NPCs viability and stimulated the senescent phenotype of NPCs, whereas MYR treatment significantly reversed SA-β-gal activity in NPCs. MYR also reduced the expression of p21 and p16 and the secretion of IL-6 and IL-8 induced by HO. RNA-seq screened 421 DEGs. The GO enrichment results showed DEGs were mainly enriched in terms such as "sterol biosynthetic process". We also found SERPINE1 has the highest log2FC abs. Silence of SERPINE1 inhibited HO-induced NPCs senescence, and overexpression of SERPINE1 could limit the anti-aging effect of MYR.

**Conclusions:**

MYR alleviated HO-induced senescence of NPCs by regulating SERPINE1 in vitro.

**Supplementary Information:**

The online version contains supplementary material available at 10.1186/s13018-022-03463-0.

## Introduction

Intervertebral disc degeneration (IDD) is an age-related degenerative disease and the main cause of various spinal degenerative diseases such as cervical spondylosis, lumbar disc herniation, and lumbar spinal stenosis. Due to the aging of the population, the prevalence of spinal degenerative diseases is increasing year by year, which not only seriously affects the quality of life of patients, but also brings a great health burden to the society [1]. However, so far the treatment of IDD has not achieved satisfactory results.

Cellular senescence is usually defined as irreversible cell cycle arrest due to replication stress and senescence [2], and the senescence-associated secretory phenotype (SASP) is an important feature of cellular senescence, which secretes a series of cytokines such as pro-inflammatory factors, growth factors, chemokines and proteases [3, 4]. The main feature of IDD is the degradation of the extracellular matrix (ECM), and NPCs, as the main functional cells of IDD, are essential to maintain the homeostasis of ECM [5]. However, senescent NPCs were accompanied by the reduction in Collagen-2, the main component of the ECM, which induced the occurrence of IDD [6], so one of the most important features of IDD is the senescence of NPCs. It has been reported earlier that the degree of cellular senescence was closely related to the IDD grade [7, 8]. Therefore, it is of great significance to explore the mechanism of senescence of NPCs for the exploration of IDD progression.

Myricetin (MYR) is a naturally occurring flavonoid compound widely found in fruits, vegetables and nuts [9]. Several studies have reported that MYR had antioxidant, antiviral, antibacterial and anticancer effects [9, 10]. Studies found that myricetin had anti-photoaging effect [11] and prevented ethanol-induced inflammatory damage [12], and could reduce the occurrence of death by inhibiting the secretion of inflammatory cytokines [13]. However, its anti-aging potential in NPCs has not been intensively investigated so far.

In this study, we identified 421 differentially expressed genes (DEGs) by RNA sequencing (RNA-seq) technology in hydrogen peroxide (HO)-induced senescent NPCs treated with MYR. The Gene Ontology (GO) database was used to enrich the DEGs into the corresponding pathways. Furthermore, we obtained the serine protease inhibitor clade E member 1 (SERPINE1) with the highest log2FC abs, which belongs to one of the serine protease inhibitor family members by Venn diagram. It was finally confirmed that MYR inhibited the senescence of NPCs by regulating the expression of SERPINE1.

## Material and methods

### Cell culture and transfection

NPCs isolated from mild IDD patients (Pfirrmann grade I–II) were a gift from First Affiliated Hospital, Xiamen University [14]. NPCs of each group were cultured in a humidified incubator with 5% CO_2_ in RPMI 1640 (Gibco, USA) supplemented with 10% fetal bovine serum (Gibco, USA) and 1% penicillin/streptomycin at 37 °C. For cell transfection, control NPCs were seeded in 6-well plates (2 × 10^5^ cells/well). When the cells reached 80% confluence, the small interfering RNA (siRNA) oligonucleotides targeting SERPINE1 (5′-GCTGACTTCACGAGTCTTT-3′) were synthesized by RiboBio (Guangzhou, China). In addition, the SERPINE1 overexpression plasmid was obtained from Sino Biological (Beijing, China), and then, Lipofectamine 3000 (Invitrogen, USA) was used to perform the transfection of the plasmids according to the manufacturer's instructions, and the transfection efficiency of the cells was verified by qPCR after 48 h of transfection at 37 °C.

### NPCs treatments

In order to screen out the most suitable HO concentration for constructing the senescent NPCs model, NPCs were treated with different concentrations of HO (10, 50, 100, 500 µM) for 24 h. In addition, NPCs were treated for 48 h with the indicated concentrations of MYR (10, 20, 40, and 80 µM) to test the cytotoxicity of MYR. HO-treated NPCs were further incubation with MYR (10 and 20 µM) for 48 h to detect the cell viability and senescent phenotype. The MYR in this experiment was purchased from Acros Organics (Thermo Fisher, Geel, Belgium).

### RNA-seq technology

RNA-seq was performed as described previously [15]. In brief, NPCs were first treated with 100 μM HO for 24 h or co-treated with 10 μM MYR for 48 h, and then, according to the manufacturer's protocol, the Illumina TruSeq RNA sample preparation kit (Illumina, Inc., San Diego, CA, USA) was used to build the sample library, and after the quality control test, the Illumina platform was used for sequencing.

### GO enrichment analysis

To reveal the biological functions of DEGs in terms of biological processes, cellular components and molecular functions, GO enrichment analysis was performed. The database (DAVID: https://david.ncifcrf.gov/) was used for GO functional enrichment analysis of DEGs, and in this study, the screening criteria for significant differential expression was set as: *P* < 0.05.

### 3-(4,5-Dimethylthiazol-2-yl)-2,5-diphenyltetrazoliumbromide (MTT) assay

To test the viability of NPCs in each group, cells were seeded at a density of 2 × 10^3^ cells/well into 96-well plates (Eppendorf, Milan, Italy) with 100 μL of medium per well, followed by the addition of MTT solution (Sigma, Milan, Italy), after 3 h of incubation at 37 °C, the supernatant was removed, and finally, dimethyl sulfoxide (DMSO; Sigma-Aldrich) was added to each well, and after 15 min of incubation with stirring, the absorbance at 490 nm was detected by a microplate reader (Thermo Fisher, Waltham, MA, USA) to determine the cells viability. The percentage of cell viability was calculated by the following formula: Cell viability (%) = Treated absorbance/Control absorbance × 100.

### Senescence-associated β-galactosidase (SA-β-Gal) staining

Each group of NPCs was seeded in six-well plates at a concentration of 3 × 10^5^ cells/well and then, stained according to the SA-β-gal staining kit method (Beyotime Institute of Biotechnology). Briefly, 1 ml of SA-β-Gal staining fixative was added to each well. After fixing at room temperature for 15 min, the cells were washed 3 times with PBS. Then, 1 ml of the prepared staining working solution was added to each well, and the stained cells were incubated at 37 °C without carbon dioxide for 12 h. Finally, the number of positive cells (magnification,  400×) in five randomly taken images was counted under a light microscope, ImageJ software (National Institutes of Health, United States) was used to measure SA-β-Gal positive cells, and the formula for SA-β-gal positive cell rate is as follows: SA-β-gal positive cell rate (%) = SA-β-gal positive cell number/total cell number × 100%.

### Quantitative polymerase chain reaction (qPCR)

Total RNA of each group of NPCs was extracted by TRIzol® reagent (Invitrogen; Thermo Fisher Scientific, Inc.) according to the manufacturer's instructions. PrimeScript RT kit (RR047A, Takara Bio Inc, Japan) was used to reverse transcribe mRNA to complementary DNA using TB Green Premix Ex Taq (RR420A, Takara Bio Inc, Japan) on a QuantStudio 5 real-time PCR system (Thermo Scientific, Wilmington, USA) for qPCR analysis. The GADPH gene was used as an internal control. The reaction program was as follows: 95 °C for 30 s, followed by 40 cycles of 95 °C for 5 s and 60 °C for 30 s. After normalization to GADPH, relative gene expression levels were analyzed by the 2^−ΔΔCt^ method. Primer sequences are presented in Additional file 1: Table S1.

### Western blot assay

Western blot was used for the expression of senescence markers P16 and P21 in NPCs in each group. According to the instructions, the total protein of each group of NPCs was extracted in RIPA lysis buffer (Millipore, Billerica, MA, USA), Equal amounts of protein were subsequently added to each well and separated by sodium dialkylsulfonate-polyacrylamide gel electrophoresis (SDS–PAGE) and then, transferred to polyvinylidene fluoride membranes (Bio-Rad Laboratories, Inc., Hercules, CA, USA). Next, the membranes were incubated with 5% bovine serum albumin for 1 h, followed by overnight incubation at 4 °C with various primary antibodies: anti-p16 (Abcam, USA), anti-P21 (Abcam, USA) and GAPDH (Ambion, Austin, TX), all primary antibodies were diluted 1:1000. Next, the membrane was washed and incubated with secondary antibody (1:8000, Bioworld technology, China) for 2 h at room temperature. Protein signals on the bands were visualized by enhanced chemiluminescence (Thermo Fisher Scientific, Waltham, USA). Protein expression levels were quantified by densitometry using ImageJ 64 software, and relative protein expression was compared to GAPDH.

### Enzyme-linked immunosorbent assay (ELISA)

The concentrations of IL-6 and IL-8 in the culture supernatants were measured by ELISA kits (Biosource International Inc.) according to the manufacturer's instructions.

### Statistical analysis

Data were statistically analyzed by SPSS 17.0 software (SPSS Inc.) and expressed as mean ± standard deviation. Student's t test was performed to analyze the differences between the two groups. *P* < 0.05 was considered to indicate a statistically significant difference.

## Results

### MYR alleviated hydrogen peroxide-induced cellular senescence of NPCs

**Fig. 1:**
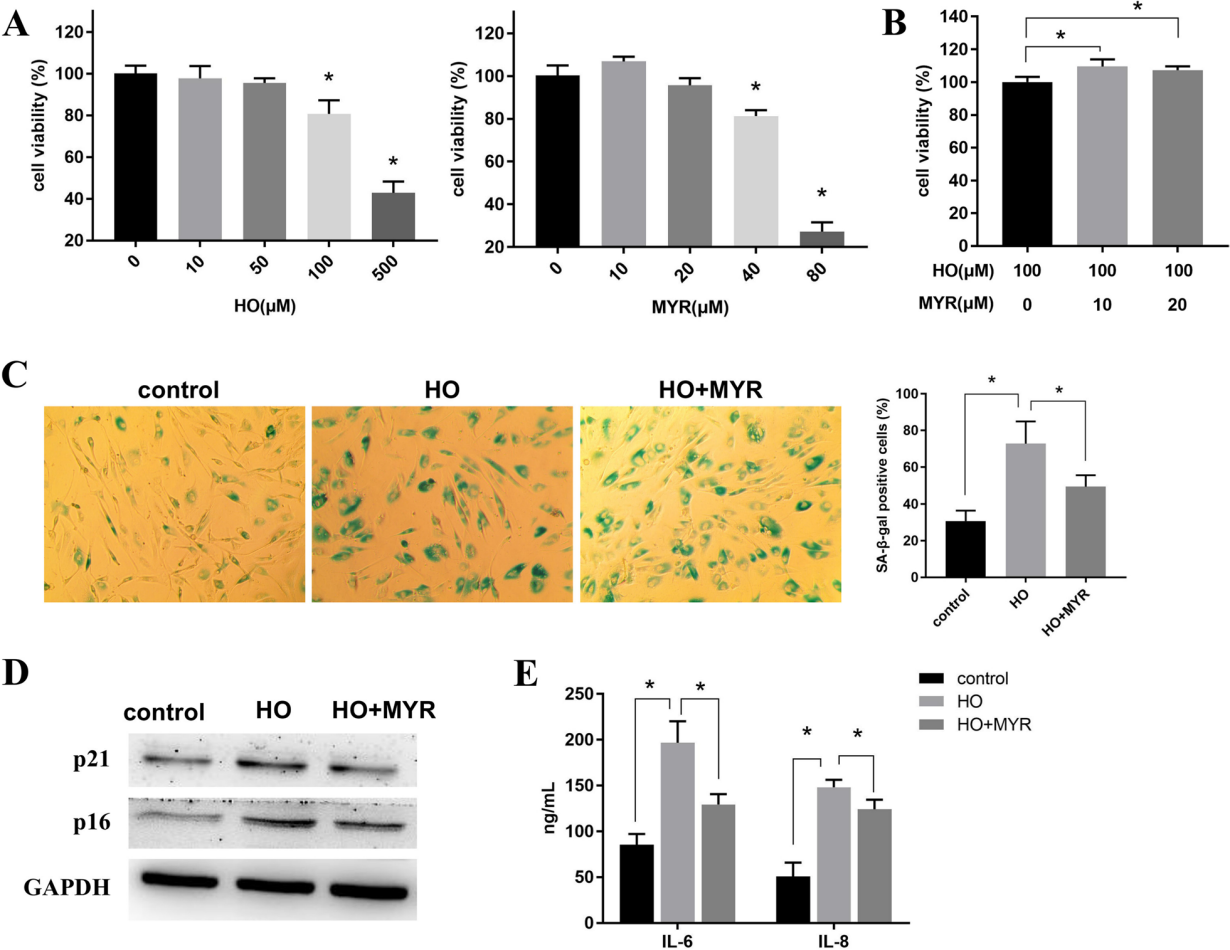
MYR inhibited HO-induced cellular senescence. **A** MTT assays were used to detect the toxicity of NPCs treated with HO or MYR. *P < 0.5 VS control group. **B** The effect of MYR on the survival of HO-treated NPCs by MTT assay. **C** SA-β-Gal staining was used to detect senescence levels of NPCs treated with HO alone or co-treated with HO and MYR. **D** Expression levels of senescence markers (p21, p16) in NPCs treated with HO alone or co-treated with HO and MYR were detected by Western blot. **E** ELISA was performed to detect the expression levels of IL-6 and IL-8 in NPCs treated with HO alone or co-treated with HO and MYR. *HO* hydrogen peroxide, *MYR* Myricetin, *NPCs* nucleus pulposus cells

First, in order to establish the senescent model of NPCs, NPCs were treated with different concentrations of HO (0, 10, 50, 100, 500 μM) for 24 h. MTT assay showed that when the HO concentration reached 100 μM, the NPCs’ viability began to decrease significantly (Fig. 1A). Next, cytotoxicity of MYR on NPCs was detected. The MTT assay revealed that, when the concentration of MYR was higher than 40 μM, the cell viability showed a significant downward trend, and MYR at 10 μM or 20 μM showed no significant cytotoxicity to NPCs (Fig. 1A). Therefore, 10 and 20 μM of MYR were used to ameliorate HO-induced growth inhibition. The results of MTT assay confirmed that MYR (10 and 20 μM) significantly enhanced the viability of NPCs treated with HO, and 10 μM of MYR showed a better effect than the dose of 20 μM (Fig. 1B).

In addition, SA-β-Gal staining showed that MYR could significantly reduce the percentage of SA-β-Gal-positive cells induced by HO (Fig. 1C). Western blot results also revealed that MYR could effectively reduce the levels of p16 and p21 of NPCs induced by HO (Fig. 1D). Finally, ELISA was used to detect the expression levels of proinflammatory SASP factors IL-6 and IL-8 in each group. The results showed that MYR showed a good inhibitory effect on the secretion of inflammatory factors induced by HO (Fig. 1D). These results demonstrated that MYR was not toxic to NPCs at concentrations below 20 μM and inhibited HO-induced senescence of NPCs when MYR was at a concentration of 10 μM.

### RNA-seq identified DEGs in senescent NPCs after MYR treatment

**Fig. 2:**
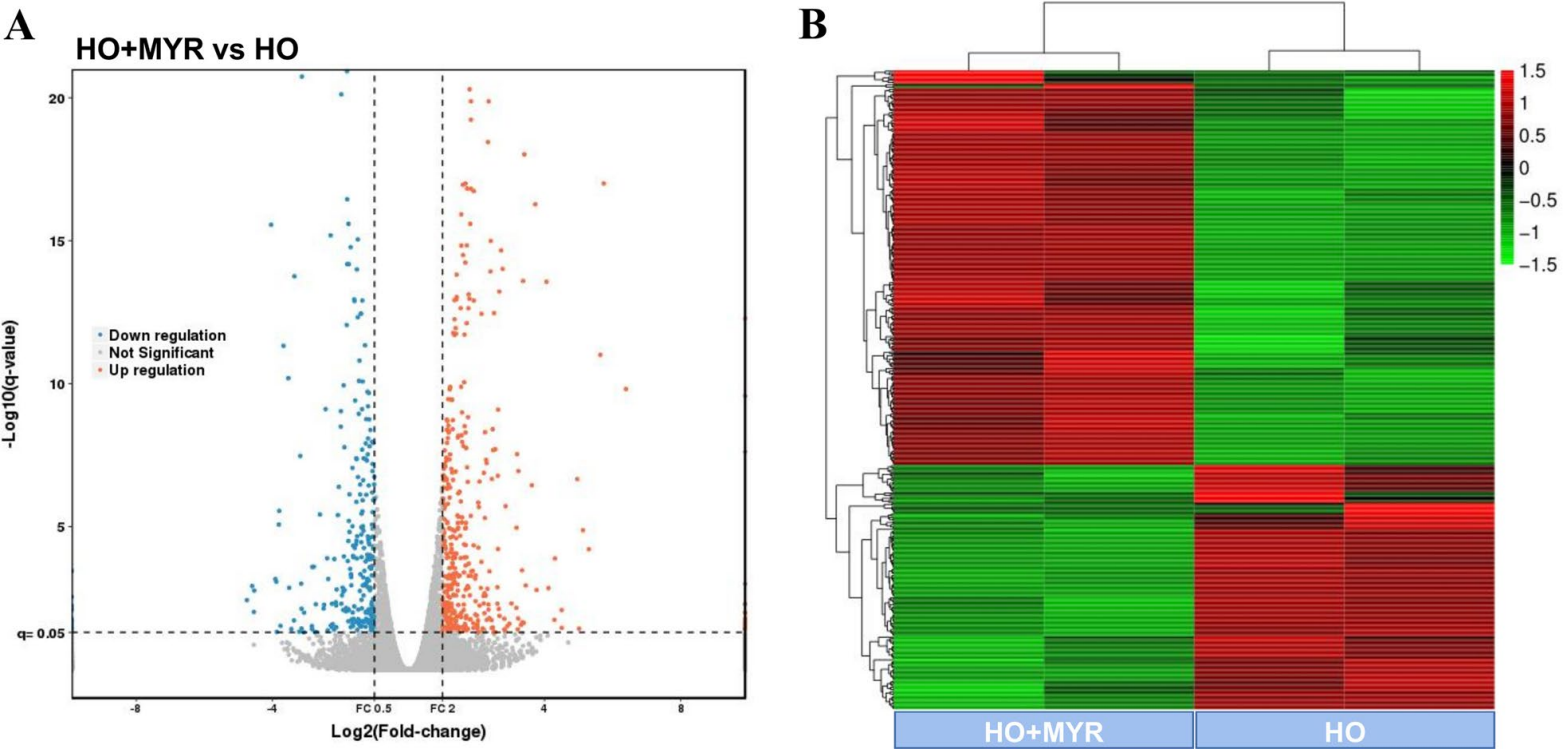
RNA-seq identified DEGs in HO-induced senescent NPCs treated with or without MYR. **A** Volcano plot of DEGs. Each point in the figure represented a DEG, red represented significantly up-regulated genes, blue represented significantly down-regulated genes, and gray represents non-significantly different genes. **B** Heatmap of DEGs. Each column represented a sample, each row represents a gene, red represents up-regulated DEGs, and green represented down-regulated DEGs

In order to explore the mechanism by which MYR affected the senescence of NPCs, HO-treated NPCs and HO + MYR-treated NPCs were used for sequencing, the volcano plot showed the most significantly different genes between the two groups (log2FC(abs) > 2 and *P* < 0.05). According to the above criteria, a total of 260 up-regulated and 161 down-regulated DEGs were obtained relative to non-MYR-treated cells (Fig. 2A, Additional file 2: Table S2). The heat-map showed an overview of the dysregulated genes in each sample (Fig. 2B). The top 10 DEGs with the largest expression changes are shown in Table 1, of which SLCO4C1 was the most up-regulated gene (log2 FC (abs) = 5.74), and AC023055.1 was the most down-regulated gene (log2 FC (abs) = 4.54).

**Table 1:** Top 10 DEGs in HO + MYR group

	Gene id	Gene name	log2FC	log2FC abs	FC abs	*P* value	Up/down	Locus
1	ENSG00000173930	SLCO4C1	5.74	5.74	53.56	1.11E−20	UP	5:102,233,986–102,296,284
2	ENSG00000151365	THRSP	5.64	5.64	49.91	3.08E−14	UP	11:78,063,861–78,068,351
3	ENSG00000268412	TRMT112P6	5.30	5.30	39.47	7.73E−07	UP	2:26,028,208–26,028,612
4	ENSG00000226428	RPL15P13	5.13	5.13	35.02	1.36E−07	UP	10:114,900,722–114,901,366
5	ENSG00000257390	AC023055.1	− 4.54	4.54	23.30	3.03E−04	DOWN	12:55,757,275–55,827,546
6	ENSG00000260537	AC012184.2	− 4.39	4.39	20.99	1.15E−49	DOWN	16:70,299,194–70,372,582
7	ENSG00000240764	PCDHGC5	4.34	4.34	20.23	2.71E−43	UP	5:141,489,081–141,512,975
8	ENSG00000203565	AL450313.1	4.32	4.32	20.00	4.19E−27	UP	10:33,684,755–33,709,868
9	ENSG00000164188	RANBP3L	4.21	4.21	18.45	9.97E−79	UP	5:36,246,913–36,302,114
10	ENSG00000263244	AC087190.3	4.12	4.12	17.34	3.04E−05	UP	16:9,104,848–9,113,181

*FC* fold change, *abs* absolute

### GO enrichment analysis of DEGs

**Fig. 3:**
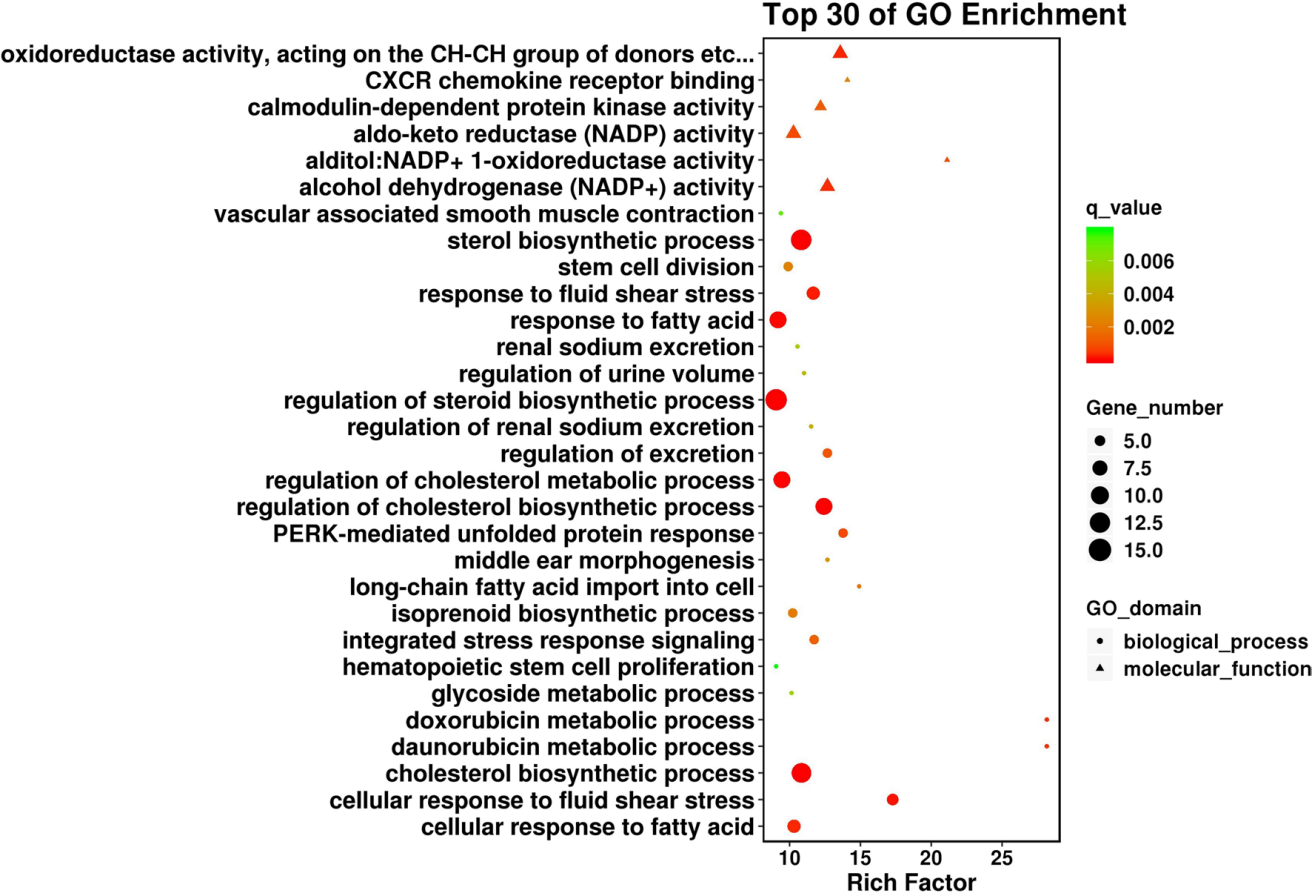
Top 30 GO enrichment analysis results for DEGs. The sizes of the circles/triangles represented the number of DEGs involved in biological processes/molecular function terms, respectively. The color of the circle/triangle indicates the *P* value, the redder the color, the more significant the difference

Next, in order to explore the function of the DEGs, GO enrichment analysis was performed, and the results showed that the biological process with the most obvious enrichment of DEGs was the "sterol biosynthesis process", and the most significant enrichment molecular function was “aldo–keto reductase (NADP) activity” (Fig. 3). In addition, the GO analysis results were searched for aging (aging, senescence) and inflammation (inflammation) related GO terms, with *P* < 0.05 as the filter condition, a total of 8 related GO biological processes were screened, as shown in Table 2.

**Table 2:** GO biological processes related to aging and inflammation

Keywords	GO_ID	GO_term	UP_GENE	DOWN_GENE	Gene_UP_list	Gene_DOWN_list	Rich_factor	*P* value
Aging	GO:0007568	Aging	13	6	FOXO4, SREBF1, ALDH3A1, JUND, PRNP, FADS1, ARG2, NFE2L2, CDKN1C, PTGS2, APOD, BCL6, EDNRB	EDN1, SERPINE1, IGFBP1, COMP, CLDN1, VCAM1	3.236	1.01E−05
Inflammat-	GO:0150077	Regulation of neuroinflammatory response	2	2	PTGS2, NR1D1	MMP3, IL6	4.370	6.03E−03
	GO:0150076	Neuroinflammatory response	2	2	NR1D1, PTGS2	MMP3, IL6	4.088	7.87E−03
	GO:0002673	Regulation of acute inflammatory response	3	1	EDNRB, PTGS2, ADORA1	IL6	3.960	8.93E−03
	GO:0002526	Acute inflammatory response	3	2	ADORA1, PTGS2, EDNRB	IL6, VCAM1	2.418	4.14E−02
	GO:0050729	Positive regulation of inflammatory response	2	4	PTGS2, AGTR1	SERPINE1, TGM2, WNT5A, IL6	2.223	4.41E−02
	GO:0006954	Inflammatory response	12	17	BCL6, AGTR1, ADORA1, CDO1, EPHA2, LPCAT3, NR1D1, EDNRB, APOD, PTGS2, NFE2L2, CD14	CXCL8, VCAM1, TGM2, CXCL6, BDKRB1, MMP3, SERPINE1, IL6, TSPAN2, CXCL5, TNFAIP3, WNT5A, NDST1, CYSLTR1, CCR1, CMKLR1, CXCL1	2.002	5.53E−04
	GO:0050727	Regulation of inflammatory response	7	6	LPCAT3, PTGS2, ADORA1, AGTR1, BCL6, EDNRB, NR1D1	SERPINE1, MMP3, IL6, TNFAIP3, TGM2, WNT5A	1.911	2.20E−02

### Silencing of SERPINE1 inhibits HO-induced cellular senescence

**Fig. 4:**
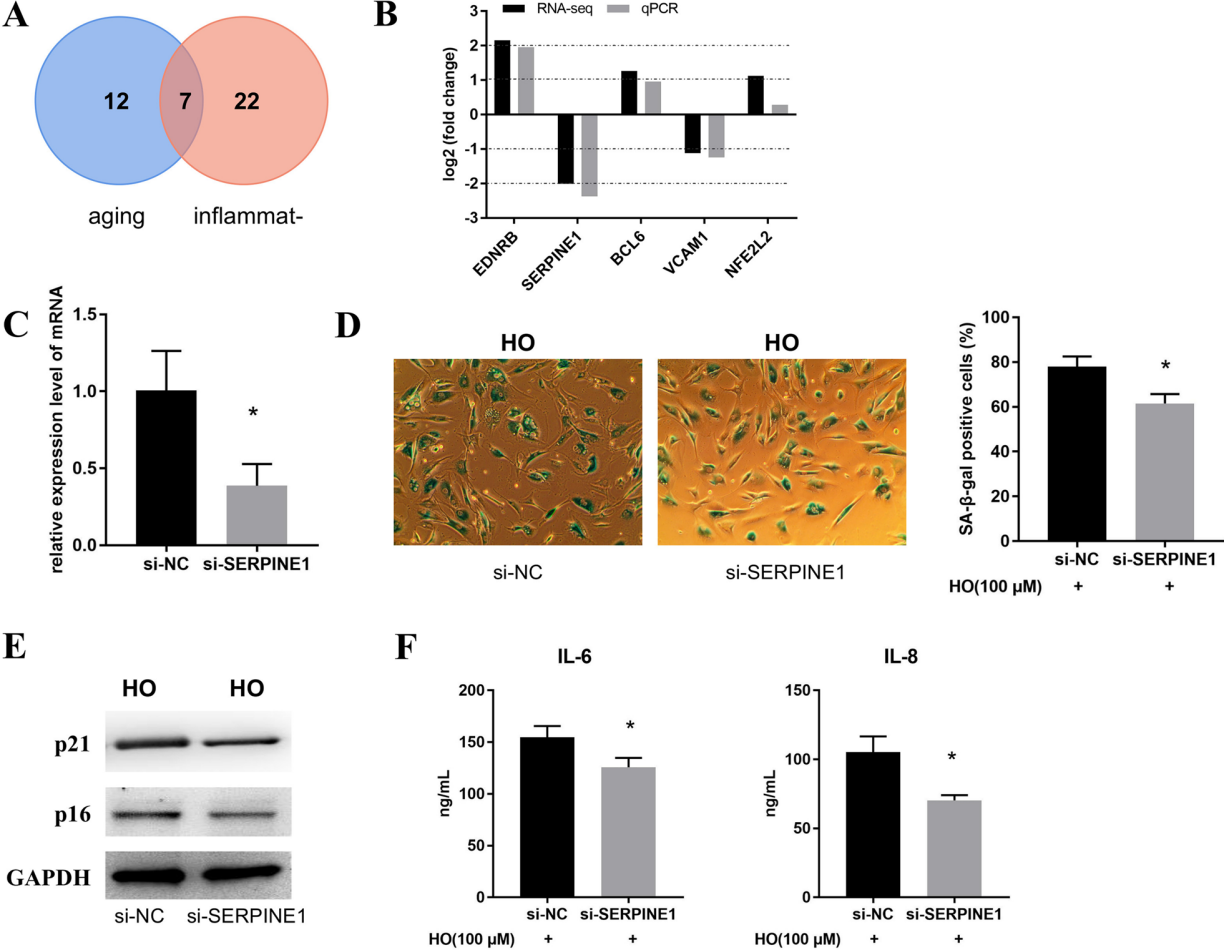
Silencing of SERPINE1 regulates HO-induced senescence in NPCs. **A** Venn diagram analysis of overlapping genes in aging and inflammation-related genes. **B** qPCR verifies the expression of the top 5 overlapping genes. **C** The silencing effect of SERPINE1 was detected by qPCR. **D** SA-β-gal staining was used to detect the level of cellular senescence in two groups of NPCs treated with 100 μM HO for 24 h. **E** Western blot was used to detect the expression levels of senescence markers (p21, p16) in HO-treated NPCs in si-NC and si-SERPINE1 groups. **F** The expression levels of IL-6 and IL-8 of NPCs treated with 100 μM HO for 24 h were detected by ELISA. *P < 0.5 VS si-NC group

Next, we first used Venn diagram to analyze the overlapping genes in aging and inflammation-related genes (Fig. 4A) and found a total of 7 overlapping genes. The expression of the top 5 overlapping genes was then verified by qPCR, and the results proved that the expression trends of these five DEGs were consistent with the RNA-seq results (Fig. 4B). More importantly, SERPINE1 was used for the following studies since SERPINE1 had the highest log2FC abs among the top 5 overlapping genes. Next, we interfered with SERPINE1 expression in NPCs, and the results proved that silencing SERPINE1 was successful (Fig. 4C). Subsequently, by SA-β-gal staining, we found that silencing SERPINE1 reduced the percentage of SA-β-Gal-positive cells (Fig. 4D). Furthermore, by Western blot, we found that silencing SERPINE1 inhibited senescence markers (p21, p16) expression (Fig. 4E). Finally, ELISA was used to detect the effect of silencing SERPINE1 on the secretion of SASP pro-inflammatory factors (IL-6 and IL-8), the results confirmed that silencing of SERPINE1 inhibited the increased expression levels of IL-6 and IL-8. Taken together, the results suggest that silencing SERPINE1 inhibits HO-induced cellular senescence.

### Overexpression of SERPINE1 inhibits the anti-aging effect of MYR

**Fig. 5:**
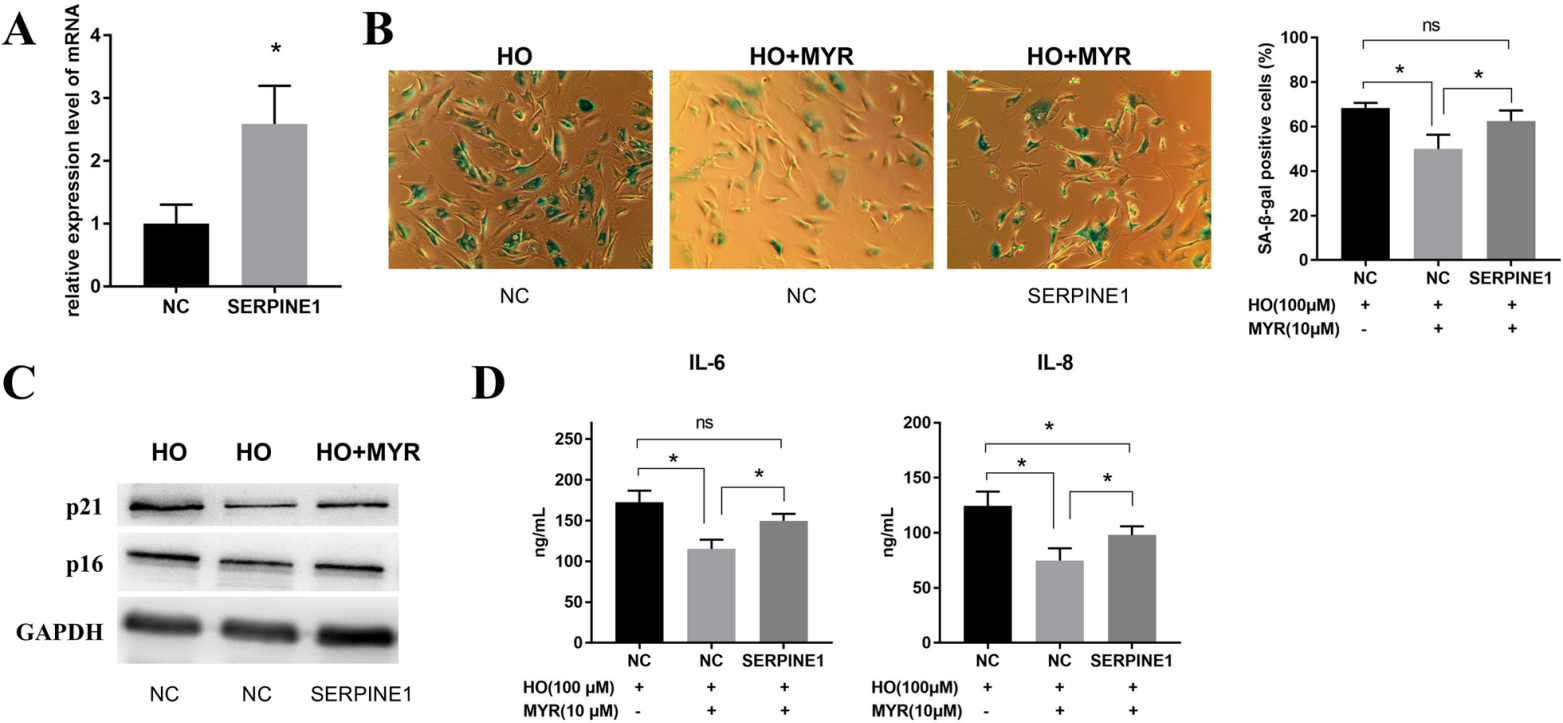
Overexpression of SERPINE1 affects the anti-aging effect of MYR. **A** qPCR was used to examine the effect of SERPINE1 overexpression. **B** SA-β-gal staining was performed to detect senescence levels in three groups of NPCs with 100 μM HO alone for 24 h or co-treated with 100 μM HO for 24 h and 10 μM MYR for 48 h. **C** Western blot was used to detect the expression levels of p21 and p16 in three groups of NPCs with 100 μM HO alone or co-treated with 100 μM HO and 10 μM MYR. **D** ELISA was used to detect the expression levels of IL-6 and IL-8 in three groups of NPCs with 100 μM HO alone or co-treated with 100 μM HO for 24 h and 10 μM MYR for 48 h

At the end of the experiment, we explored the effect of SERPINE1 on the anti-aging effect of MYR. First, we verified the transfection efficiency of SERPINE1 overexpression in NPCs. qPCR results showed that SERPINE1 was overexpressed successfully (Fig. 5A). Subsequent SA-β-gal staining (Fig. 5B) and Western blot (Fig. 5C) results confirmed that overexpression of SERPINE1 reversed the MYR-induced decrease in the percentage of SA-β-Gal-positive cells and expression of p16 and p21. Similarly, ELISA results showed that overexpression of SERPINE1 inhibited the expression of MYR-regulated IL-6 and IL-8 (Fig. 5D). Taken together, the results indicated that overexpression of SERPINE1 inhibited the anti-aging effect of MYR.

## Discussion

IDD is one of the ancient and common clinical diseases, and cellular senescence is the key inducement of IDD pathogenesis. In the present study, we found that MYR could effectively inhibit HO-induced cellular senescence and the secretion of inflammatory factors of NPCs by regulating SERPINE1.

The causes of cellular senescence are mainly divided into two categories, replicative aging and stress-induced premature aging. The former is a programmed death process, while the latter is under the action of some sub-lethal emergencies such as hyperoxia, HO, ultraviolet rays cells age in advance. HO can directly damage DNA and cause premature cell aging [16], so in this study HO was selected as an agent for inducing senescence in NPCs. The concentration of HO used for aging induction of NPCs was not consistent in previous studies [17–19], and the major considered parameter was the cell viability, which was generally at 80–90% [17, 18]. Consistently, the concentration (100 μM) we used for aging induction resulted in a ~ 80% viability of NPCs. With the increase in HO dose, cell viability reduced gradually, and only 40% of NPCs was alive after treated with 500 μM HO. Generally, the concentration of HO caused ~ 50% of cell viability was used to induce apoptosis of NPCs [20, 21].

MYR is a flavonol compound with various pharmacological activities such as anti-inflammatory and analgesic, anti-tumor, hypoglycemic, and liver protection [9]. MYR shows abundant resource advantages and huge potential utilization value. Multiple effects of MYR have been reported to depend on the therapeutic dose [22]. Cell viability assay indicated that the higher dose of MYR (40 and 80 μM) significantly inhibited cell growth, while the lower dose groups (10 and 20 μM) showed no cytotoxicity on NPCs. We also found that lower dose of MYR (10 and 20 μM) could not significantly promote the growth of NPCs, but they played positive growth effects on HO-treated NPCs. We inferred that the injured NPCs could not growth as normal, and the potential anti-aging and anti-inflammation effects of MYR helped the injured NPCs to gradually return to a better state. In addition, the positive growth effect of MYR on normal NPCs may be observed by extending the treatment time to 72 h, as a higher viability was observed in 10 μM group treated for 48 h. Further senescent phenotype detection results indicated that MYR could inhibit the senescence of NPCs.

Inflammatory response has always been an important factor affecting the occurrence and development of various degenerative diseases including IDD. It is difficult for the normal immune inflammatory response to act on the intervertebral disc tissue under physiological conditions. However, when disc herniation occurs, herniated disc tissue causes pain responses through the action of inflammatory factors [23]. Previous studies have demonstrated that MYR could inhibit the expression of inflammatory factors [13]. Consistently, we found that MYR inhibited the expression of inflammatory factors IL-6 and IL-8 secreted by HO-induced senescent NPCs.

Next, RNA-seq was used to further explore DEGs in senescent NPCs to uncover the key genes affecting NPC aging. We found a total of 260 up-regulated genes and 161 down-regulated genes compared with the control group. In the GO enrichment analysis of DEGs, DEGs were mainly enriched in the "CXCR chemokine receptor binding" terms, a previous report demonstrated that loss of CXCR7 expression leads to cellular senescence [24]. It indicated that DEGs may play a role by participating in CXCR chemokine receptor binding. At the same time, we searched the GO terms related to aging (aging, senescence) and inflammation (inflammat-), respectively, and a total of 8 related GO biological processes were screened. FOXO4 was enriched in the GO term of "aging", and it has been proved to be involved in IDD [25, 26]. In the term of "inflammatory response", the members of C-X-C motif chemokine ligand (CXCL) family, including CXCL1, CXCL, CXCL5, CXCL6, and CXCL8, were downregulated after MYR treatment. In addition, matrix metalloproteinases (MMPs), playing a positive role in matrix degradation, have been considered to be an important guiding factor in IDD, and overexpression of them has been shown to exacerbate a variety of degenerative diseases including osteoarthritis [27]. GO enrichment analysis indicated that MMP3 was downregulated after MYR treatment. Therefore, we inferred that MYR might also play a positive role in ameliorate matrix degradation ability of senescent NPCs, which also waiting for further investigation.

We continued to analyze the overlapping genes in senescence and inflammation-related genes by Venn diagram, and screened out 7 DEGs. The log2 FC (abs) value of the 7 DEGs varied from 1.02 to 2.15, so only the top 5 DEGs (EDNRB, SERPINE1, BCL6, VCAM1 and NFE2L2) with higher log2 FC (abs) value were selected for verification. The result indicated that SERPINE1 had the highest log2 FC (abs) value among the five candidates, thus attracting our attention. SERPINE1 belongs to the serine protease inhibitor family [28] and has been found to show pro-angiogenic, growth and migration stimulation and anti-apoptotic activities in recent years [29]. It is also confirmed to be the most reliable biological and prognostic marker for various cancers [30, 31]. More importantly, it is reported that SERPINE1 has showed important regulating role in aging [32, 33] and is also able to regulate inflammatory damage [34], but its role in the senescence of NPCs is still unknown. In this study, we found that the expression of SERPINE1 was significantly decreased in senescent NPCs cotreated with HO and MYR, and interfering with SERPINE1 expression could inhibit the secretion of inflammatory factors in NPCs, which was consistent with previous reports [34]. In addition, studies have shown that SERPINE1 can promote the expression of STAT3 signaling pathway [35], and studies have found that the aging of NPCs was closely related to STAT3 signaling pathway [36]. Therefore, we speculated that SERPINE1 may regulate the aging of NPCs by regulating the STAT3 signaling pathway.

qPCR validation also indicated that EDNRB’s validated log2 FC (abs) value was second only to that of SERPINE1. It is reported that EDNRB is closely correlated with hair graying with aging [37], and deletion of EDNRB leads to delayed development of neural crest cells [38]. Another noteworthy verified DEG is BCL6, which has been shown to regulate cellular senescence [39]. In addition, BCL6 is reported to be a potent inhibitor to suppress the senescence of mouse fibroblasts, and it could induce cyclin D1 expression thus bypassing the senescence response downstream of p53 [40]. The upregulation of the two candidates might also be involved in the anti-aging effect of MYR, which is worth for further studies.

Overall, the present study found that MYR was able to alleviate HO-induced senescence of NPCs by regulating the expression of SERPINE1 in vitro*,* providing a promising candidate molecule to reverse the senescence of NPCs in vivo. The study also identified and verified other candidate DEGs in MYR treating group, which helps to further investigate the multiple mechanisms of MYR.

## Supplementary Information

**Additional file 1**. Table S1: Primer sequences used for qPCR.

**Additional file 2**. Table S2: Fold change of all 421 DEGs.

## Data Availability

The datasets used and/or analyzed during the current study are available from the corresponding author on reasonable request.
